# Effectiveness of an Electronic Partogram: A Mixed-Method, Quasi-Experimental Study Among Skilled Birth Attendants in Kenya

**DOI:** 10.9745/GHSP-D-19-00195

**Published:** 2019-12-23

**Authors:** Harshadkumar Sanghvi, Diwakar Mohan, Lindsay Litwin, Eva Bazant, Patricia Gomez, Tara MacDowell, Levis Onsase, Valentino Wabwile, Charles Waka, Zahida Qureshi, Eunice Omanga, Anthony Gichangi, Ruth Muia

**Affiliations:** a Jhpiego, Baltimore, MD, USA.; b Johns Hopkins Bloomberg School of Public Health, Baltimore, MD, USA.; c University of Nairobi, Nairobi, Kenya.; d Kenya Ministry of Health, Nairobi, Kenya.

## Abstract

**Background:**

Timely identification and management of intrapartum complications could significantly reduce maternal deaths, intrapartum stillbirths, and newborn deaths due to hypoxia. The World Health Organization (WHO) identifies monitoring of labor using the paper partograph as a high-priority intervention for identifying abnormities in labor and fetal well-being. This article describes a mixed-method, quasi-experimental study to assess the effectiveness of an Android tablet-based electronic, labor clinical decision-support application (ePartogram) in limited-resource settings.

**Methods:**

The study, conducted in Kenya from October 2016 to May 2017, allocated 12 hospitals and health centers to an intervention (ePartogram) or comparison (paper partograph) group. Skilled birth attendants (SBAs) in both groups received a 2-day refresher training in labor management and partograph use. The intervention group received an additional 1-day orientation on use and care of the Android-based ePartogram app. All outcomes except one compare post-ePartogram intervention versus paper partograph controls. The exception is outcome of early perinatal mortality pre- and post-ePartogram introduction in intervention sites compared to control sites. We used log binomial regression to analyze the primary outcome of the study, suboptimal fetal outcomes. We also analyzed for secondary outcomes (SBAs performing recommended actions), and conducted in-depth interviews with facility in-charges and SBAs to ascertain acceptability and adoptability of the ePartogram.

**Results:**

We compared data from 842 clients in active labor using ePartograms with data from 1,042 clients monitored using a paper partograph. SBAs using ePartograms were more likely than those using paper partographs to take action to maintain normal labor, such as ambulation, feeding, and fluid intake, and to address abnormal measurements of fetal well-being (14.7% versus 5.3%, adjusted relative risk=4.00, 95% confidence interval [CI]=1.95–8.19). Use of the ePartogram was associated with a 56% (95% CI=27%–73%) lower likelihood of suboptimal fetal outcomes than the paper partograph. Users of the ePartogram were more likely to be compliant with routine labor observations. SBAs stated that the technology was easy to use but raised concerns about its use at high-volume sites. Further research is needed to evaluate costs and benefit and to incorporate recent WHO guidance on labor management.

**Conclusion:**

ePartogram use was associated with improvements in adherence to recommendations for routine labor care and a reduction in adverse fetal outcomes, with providers reporting adoptability without undue effort. Continued development of the ePartogram, including incorporating new clinical rules from the 2018 WHO recommendations on intrapartum care, will improve labor monitoring and quality care at all health system levels.

## Abstract (precis)

Use of the electronic partogram, a digital labor-support application, is associated with improved fetal outcomes and greater use of interventions to maintain normal labor compared to the paper partograph.

## INTRODUCTION

In 2015, the World Health Organization (WHO) estimated that 303,000 maternal deaths occur worldwide each year.^1^ A 2013 global burden of disease study estimated that 6.4% of maternal deaths annually were due to obstructed labor.^2^ In addition, 1.3 million intrapartum stillbirths and 904,000 newborn deaths due to hypoxia occur each year.^3^^,^^4^ Timely identification and management of intrapartum complications could prevent many of these deaths.^5^ With the global impetus toward universal health coverage, more women are choosing to give birth in health facilities; however, health outcomes will not improve unless service quality is assured.^6^ WHO identifies monitoring of labor to guide timely, appropriate actions as a high-priority quality improvement intervention.^7^

Labor management requires skilled birth attendants (SBAs) to record periodic observations of maternal and fetal well-being, use these data to distinguish normal from abnormal progress, and predict and plan next steps over the course of labor. Interpreting a single measurement such as fetal heart rate is relatively simple, but evaluating combinations of measurements (e.g., labor progression in relation to frequency and duration of contractions) is complex. In optimal labor management, women progressing normally are supported by ambulation, oral fluids, feeding, and presence of a companion of choice, and unwarranted use or overuse of interventions, such as artificial rupture of membranes and augmentation, are avoided. When labor abnormalities occur, appropriate actions are taken.

The paper partograph is the most commonly available labor-monitoring tool, used by health professionals and recommended by WHO for active labor.^8^ The WHO partograph is a graphic representation of measures of fetal and maternal well-being and labor progression that facilitates identification of obstetric and fetal complications. Routine use of the paper partograph in low- and middle-income countries is inconsistent,^9^^,^^10^ and in many settings, SBAs complete partographs retrospectively for recordkeeping purposes only.^11^^–^^14^

A study in Kenya in 2010 found that “the partograph was widely used during labor (in 88% of 442 cases) and was initiated at the correct time in more than 90% of the cases; however, all components were completed in only 58% of cases.”^15^ A 2018 Cochrane review concluded that the quality of existing evidence was too low to determine whether using a paper partograph compared to nonuse of a paper partograph affected the rate of cesarean delivery or incidence of low Apgar scores.^16^ Using a partograph may make little difference in labor length (low-quality evidence) or the number of women who receive oxytocin to accelerate labor (moderate-quality evidence).^16^ Bedwell et al. concluded from a realist review that although the paper partograph appears to be accepted, evidence suggests that it is not being used in practice as anticipated and thus is not reaching its potential in improving outcomes.^17^

Several developers have focused on low-cost digital applications to address deficiencies in the paper partograph, improve recordkeeping, support decision making, and enhance quality of care during labor and delivery.^18^^–^^21^ In one of the first published evaluations of digital labor-support applications, Litwin et al. reported on the use of an Android tablet-electronic partogram application (ePartogram) in Zanzibar.^22^ Health workers quickly became competent and confident in using the ePartogram application on a tablet and believed its use improved timeliness of care and supported decision making.

This study seeks to assess the effectiveness of ePartogram use on health outcomes in limited-resource settings and to ascertain the acceptability and adoptability of the ePartogram based on health workers’ experiences.

## METHODS

### Study Design

This was a mixed-method, quasi-experimental evaluation of labor management outcomes. Our intervention included a 2-day refresher training for SBAs and supervisors in control and intervention sites and introduction and training on use of the ePartogram in intervention sites. All outcomes except one were during the intervention period, comparing the ePartogram intervention. The exception was outcome of early perinatal mortality before and during ePartogram introduction in intervention sites compared to control sites.

### Study Setting and Sites

The study was conducted from October 2016 to May 2017 in 12 health facilities serving 2 counties, Kisumu in western Kenya and Meru in eastern Kenya. The 2 tertiary care facilities, 1 per county, were allocated to receive the intervention. The other facilities were selected after matching on characteristics like birth volume, staffing level, facility type (public or private), and provision of basic and/or comprehensive emergency obstetric and newborn care (BEmONC and CEmONC, respectively). We allocated 2 large CEmONC facilities to the ePartogram group. The remaining 10 facilities had similar overall delivery rates and were randomly allocated to the intervention or comparison group. Thus, the ePartogram group had 2 large CEmONC and 4 BEmONC facilities, and the paper partograph group had 4 small CEmONC and 2 BEmONC facilities. The Kenyan public health care structure has large central tertiary hospitals, usually 1 per county, and smaller district hospitals and health centers. The larger hospitals usually have a specialist obstetrician-gynecologist and many midwives, and health centers often only have 1 or 2 midwives. Often care is given by providers who do not fit the WHO definition of SBAs. Public services are complemented by faith-based institutions and private maternities.

Kenyan health facilities use the WHO modified partograph with alert and action lines; a partograph is started once the woman’s cervix is 4 cm dilated. SBAs are supposed to fill out partographs, but many Kenyan health facilities are poorly staffed, and sometimes partographs are completed by nurses, students, and other non-SBAS who are deployed in labor wards.

### The Intervention

**FIGURE 1.**
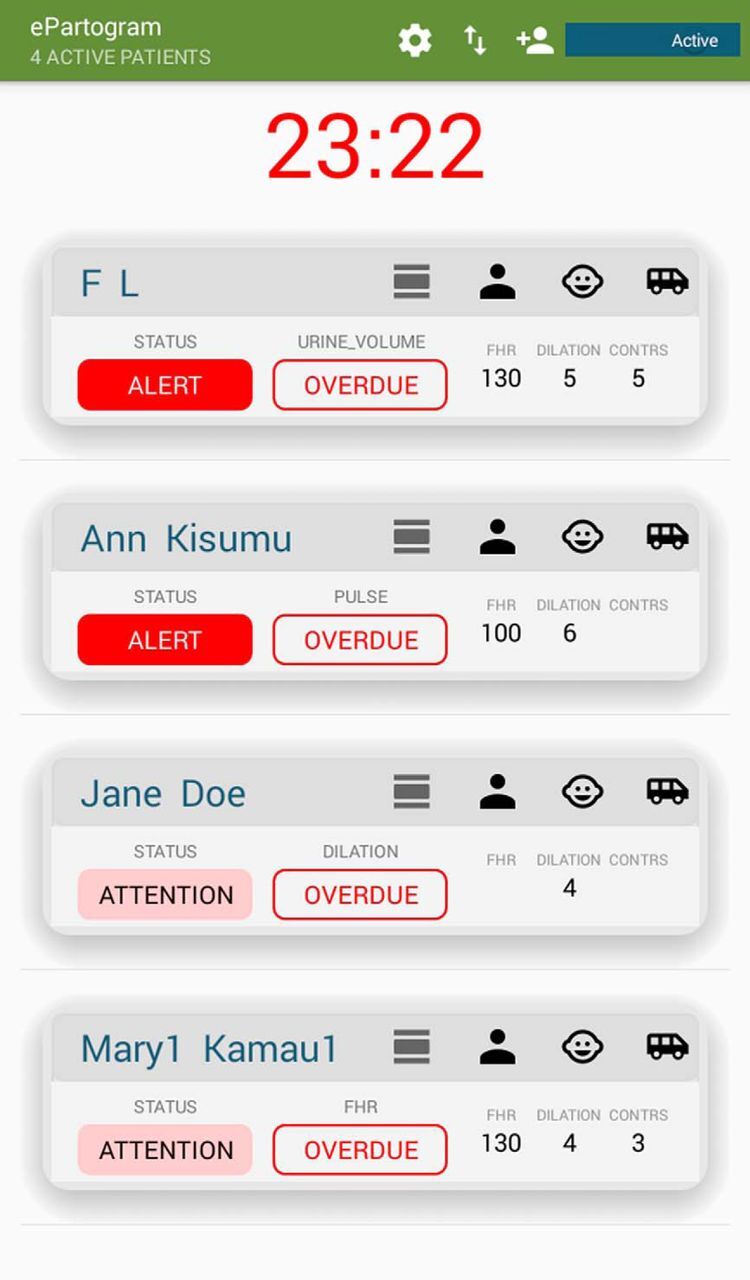
ePartogram Dashboard Screenshot Dashboard shows patients under care of a provider, alerts, and overdue assessments.

The ePartogram is an Android tablet-based application developed using human-centered design between 2011 and 2017 to address many challenges of monitoring labor with the paper partograph. The ePartogram is a clinical decision-support tool with algorithms and clinical rules that are based on WHO guidance for managing normal and complicated labor. The app provides auditory reminders to prompt providers to take measurements when due and provides visual and auditory alerts when clinical rules are triggered that predict complications (via low-level alerts) or detect complications (via high-level alerts) (Figure 1). The app allows for increased data entry efficiency by automatically graphing data and storing all client files within the application. In addition, retrospective entry of data is prevented. The decision-supporting software is based on 77 clinical rules based on measures of fetal and maternal well-being, progress of labor, and expected trends as labor progresses.

The ePartogram app was developed to address many challenges of monitoring labor with the paper partograph and improve decision making.

The ePartogram app can handle multiple patients who a provider may be taking care of. A dashboard displays all the patients in a provider’s care and highlights those that need immediate attention. In intervention sites, supervisors had access to digitally transmitted ePartogram data anytime on a tablet device if they chose to review and act on them. In control sites, supervisors had access to paper partographs when they visited or were called to the labor ward, which was the supervision norm. Remote supervision off-site with the ePartogram was possible, but we did not activate this function during the study. Data were stored securely in the cloud and were available in real time. The app also prompted the provider to record actions and interventions they took. The app can be tailored to the needs of the country by adding or removing clinical rules. The technical details of the ePartogram are described elsewhere.^22^

The usability and functionality of the ePartogram underwent field validation exercises in Kenya and Tanzania and a feasibility study in Zanzibar.^22^ Qualitative feedback from the Zanzibar study informed additional functional enhancements to the ePartogram, including the ability to print a paper record of the ePartogram, inclusion of a dashboard for supervisors and managers, the ability to digitally transfer the full labor record to referral sites, and use of more intuitive icons on the app.

### Participant Recruitment, Consent, and Training

Study coinvestigators trained 9 master trainers during a 2-day workshop. This training-of-trainers workshop included interactive presentations and case studies, and it facilitated practice using both the paper partograph and ePartogram. Clinicians meeting the WHO definition of SBA (that is, a doctor, nurse, or midwife “trained to proficiency in the skills needed to manage normal [uncomplicated] pregnancies, childbirth and the immediate postnatal period, and in the identification, management and referral of complications in women and newborns”^23^) and caring for women in active labor at study sites were recruited. We did not include midwifery students or any unqualified birth attendant even though they often provide labor care in these facilities. All SBAs in a selected facility were recruited to the same study group and gave individual written consent to participate.

All participating SBAs and supervisors completed a 2-day labor management update conducted by master trainers and coinvestigators. This training included case studies on using the paper partograph, decision making, and managing normal labor and common labor complications according to WHO and Kenya Ministry of Health guidelines. Additional topics included respectful maternity care; recognition and management of fetal, maternal, and labor progression abnormalities; fetal distress; pre-eclampsia/eclampsia; and fever. Trainees were also refreshed on when supervisors should be contacted in response to labor abnormalities identified, the standard operating procedures for handing over patients as shifts changed, and how to fill out facility birth and outcome registers. We had an additional layer of scrutiny for the ePartogram group only, although this did not involve helping with clinical issues and dealt only with fixing the occasional technology-related issues, such as failure of supervisor tablets to sync with SBA tablets.

All participating skilled birth attendants and supervisors completed a 2-day labor management update.

**FIGURE 2.**
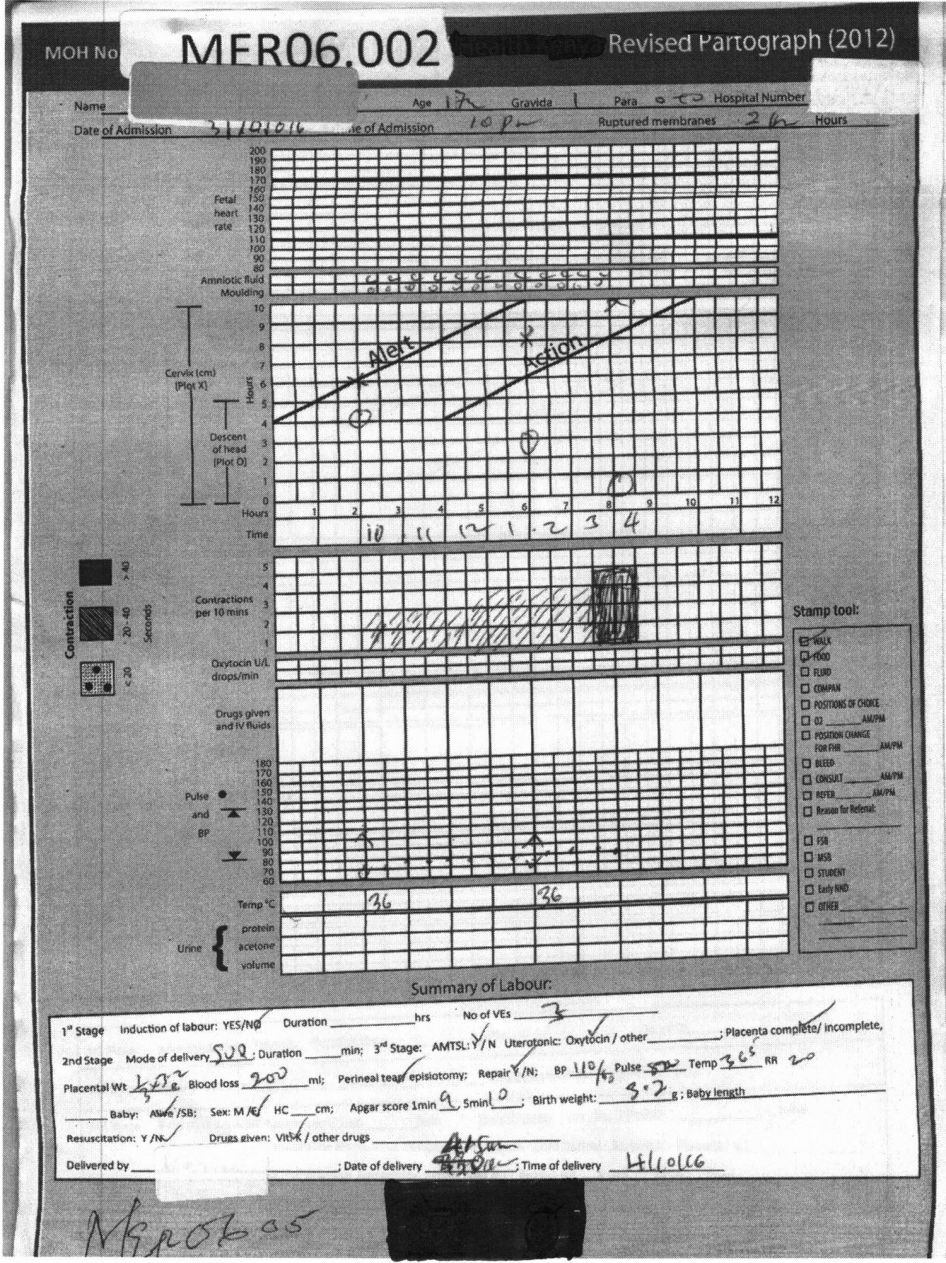
Completed Paper Partograph With Stamp Tool to Record Interventions

We used guided case studies to instruct SBAs on how to interpret clinical information displayed on the partograph. The content and approach for the 2-day labor management training was identical for both study arms. The ePartogram provided prompts to record interventions and actions that the SBA takes, and because this was not available on the paper partograph, we included an action-taken sticker on all paper partographs so that actions could be recorded (Figure 2).

Intervention group participants were also trained in the use of the ePartogram application, maintenance of the tablets, and handover protocols. During the day-long ePartogram training, we provided step-by-step didactic training on the application. We also trained the participants on the standard operating procedures for ePartogram use, storage, and cleaning and on how to print from the ePartogram.

We excluded SBAs who did not pass the written assessment during the update training, did not pass the written or skills-based assessments, or did not complete the training. Overall, 72 SBAs were trained in the intervention group, of whom 69 passed the assessments and consented to participate the study. Of the 42 SBAs trained in the comparison group, 1 withdrew from the study. At all study sites, women in active labor received care according to global and national standards. SBAs in intervention facilities used the ePartogram to document measurements; back-up paper partographs were available at each site. In the comparison facilities, SBAs used paper partographs to monitor women in labor. Client names and identifiers from the ePartograms and paper partographs were removed before partographs were scanned. We only used the partographs and labor records filled out by trained, consenting providers for this study.

### Outcomes

Study outcomes relate to compliance with globally recommended labor-monitoring practices and recording the measurements obtained on the paper partograph and ePartogram; actions taken to maintain normal labor; detection of and actions taken to address deviations from normal labor and complications; and client outcomes. Table 1 describes the indicators used as outcome measures.

**TABLE 1.** Outcome Measures Used in the Mixed-Method Quasi-Experimental Study of the Effectiveness an Electronic Partogram

**Primary Outcome Measure**
1. Percentage of ePartogram/partographs showing fetus/newborn with a suboptimal fetal/newborn outcome (defined by presence of fresh stillbirth; newborn Apgar score of 5 or below at 1 minute, or 7 or below at 5 minutes; or newborn resuscitation needed) as recorded on the ePartogram/partograph by the SBA.
**Secondary Outcome Measures**
1. Percentage of ePartogram/partographs with a suboptimal maternal outcome (defined by presence of retained placenta, blood loss greater than or equal to 500 ml, systolic blood pressure less than 90 mm Hg or equal to/greater than 140 mm Hg, diastolic blood pressure less than 60 mm Hg or equal to/greater than 90 mm Hg, and pulse less than 60 beats per minute or equal to/greater than 100 beats per minute) as recorded by the SBA on the ePartogram/partograph.
2. Percentage of ePartogram/partographs with any action recorded on the ePartogram/partograph to maintain normal labor, among all partographs or ePartograms. Actions to maintain normal labor include ambulation and encouragement to eat or drink.
3. Percentage of ePartogram/partographs with any action recorded on the ePartogram/partograph to address any sign of deviation from normal labor, among all paper partographs or ePartograms to support detection, decision making, and action to address deviations from normal labor and complications arising during labor. Actions included providing oxygen, changing the position of the laboring woman in response to fetal heart rate abnormalities, checking for bleeding, consulting with a supervisor, referring a client to another facility, and augmenting labor during the first stage.
4. Incidence of fresh stillbirth and neonatal death within 24 hours among all births in a month, according to aggregate monthly routine facility data recorded on facility registers by SBAs.

Abbreviations: ePartogram, electronic partogram; SBA, skilled birth attendant.

### Study Sample Size

When the study was originally conceived, the sample size was calculated at 2,600 based on detecting differences in outcomes among laboring women whose parameters placed them to the right of the alert line (denoting abnormal progress). As the action line has in recent times been increasingly called into question, we abandoned a sample size based on that parameter. In addition, recruitment was disrupted by a health worker strike, resulting in a significantly larger proportion of patients arriving late in labor. Hence, we have presented a power analysis of the final effective sample size.

Overall, 1,884 paper partographs and ePartograms were collected. Analysis was done on a total sample of 1,884 and on subsets of this sample where some variables were missing. For some outcomes, we restricted the study analysis to those with 2 or more entries, giving a sample of 1,609. For fetal outcomes, after accounting for missing data, we analyzed a final sample size of 1,405. We performed a post hoc calculation using a type I error of 0.05 and power of 80% to assess the detectable difference. The primary outcome was a composite fetal outcome with a control group prevalence of 18% and an intraclass correlation coefficient of 0.11. The sample size would have been sufficient to detect a 16% difference between the intervention and comparison groups.

### Quantitative Data Collection and Extraction

A trained health records information officer at each facility de-identified and scanned each completed partograph into the study database for cleaning and analysis. All paper partographs had an additional data collection tool stamped on them (Figure 2) or integrated into the electronic app (ePartogram), which asked whether the SBA instructed the client to walk/ambulate, eat food, or drink water during labor; gave the client fluid; encouraged the presence of a birth companion; administered oxygen; changed the client’s position; checked for bleeding; consulted a specialist; referred the client to another site; or performed any other clinical action. Data were also collected about the number of births and maternal and newborn outcomes reported monthly in the maternity ward register. Once research assistants and/or study staff members received these files, they checked them to ensure they were legible, scanned correctly, and titled correctly and then saved the files in a master folder. Research assistants reviewed these scanned partographs and extracted data into the clinical and translational research software, Research Electronic Data Capture (REDCap).^24^ Research assistants were not blinded to use of the ePartogram/partograph. Each research assistant’s data extraction was validated by a sample of charts that were double entered by another assistant as part of the extraction training process. All data were abstracted by the researchers using REDCap.

### Analysis of Individual Partographs

Data were analyzed in Stata version 14.2.^25^ For this study, we assumed that missing data in paper partograph and ePartogram measurements were not missing at random; hence, we did not impute them. All estimates presented were based on complete case analysis. We used frequencies and percentages for categorical variables and median to present descriptive statistics for client and facility characteristics across the study groups. We tested hypotheses using nonparametric methods with Fisher exact tests for categorical variables and the test of medians for continuous variables. To assess the intervention’s overall impact, we created a log binomial model with fetal outcome as the dependent variable and ePartogram use as the independent (intervention) variable. We used facility and individual level variables as control variables as part of multivariable regression model to account for the baseline differences between the intervention and comparison groups. The control variables included the type of facility (CEmONC versus BEmONC), affiliation (public versus faith-based), and number of providers at the facility. We also controlled for a nationwide health worker strike that disrupted health services at study sites between December 2016 and February 2017. At the individual level, we used parity (primipara versus multipara) and cervical dilation at admission (≤5 cm versus >5 cm). The adjusted relative risks are presented as the effect size of the ePartogram intervention along with their 95% confidence intervals (CIs). Because laboring women may have been attended to by multiple providers from the facility, we accounted for clustering only at the level of the facilities using Huber-White sandwich estimators.

### Analysis of Facility Registers

To estimate the intervention’s effect, we compared data obtained from facility registers from 6 months before the study (May–October 2016) and after the implementation of the study (May 2017). All SBAs and supervisors were updated on how to correctly fill out facility registers during the training. In addition, register data were extracted and collected weekly from study-trained health records officers. No other effort was made to ensure completeness or validity of these data. The data points included the monthly aggregates for total births, number of stillbirths, and number of newborn deaths in the first 24 hours for each facility. The final analysis included register data from the 12 facilities on births over 12 months (2 facilities had 0 births in a month). The outcome variable was early perinatal mortality, defined as early neonatal (<24 hours) death and fresh stillbirth over a denominator of the sum of live births and fresh stillbirths. We analyzed for difference between intervention and comparison groups before and after the study by fitting a population-averaged generalized linear model using generalized estimating equations with a Poisson distribution, log link, and exchangeable correlation structure. The models were adjusted using facility-level clustering and adjusts for facility variables of affiliation (public versus private), capacity to address emergencies (CEmONC versus BEmONC), presence of a medical or clinical officer (yes/no), percentage of providers trained in labor management by study staff (≥75% or <75%), median years of provider experience, and whether a health worker strike was occurring in the month in public facilities (yes/no). Incident rate ratios (IRRs) with 95% CIs for before and after rates in each study group are presented along with the difference-in-difference estimator (ratio of IRRs).

### Qualitative Data Collection and Analysis

Four facility in-charges and 28 SBAs from both arms participated in in-depth interviews lasting 30 to 35 minutes. Interviews were conducted in English, recorded, and transcribed. Data collectors were implementing agency staff trained in qualitative research data collection procedures using a data collector training guide^26^ and study ethics. We coded the qualitative data in Atlas.ti version 7 software,^27^ and created a codebook based on field guide topics and themes that emerged from the interviews. Interview data saturation was deemed to have been reached, as no new themes emerged in the final interview and all themes were mentioned to some extent in all interviews. The analysis followed the framework analysis process recommended by Ritchie et al.^28^ We identified themes and subthemes to align with research questions; we describe these later using illustrative quotations.

### Ethical and Safety Considerations

The study was approved by the Johns Hopkins Bloomberg School of Public Health Institutional Review Board (JHSPH IRB #6958) and the Kenya Medical Research Institute (KEMRI protocol #530). SBAs gave informed written consent before participation. Women cared for using the ePartogram did not have a paper partograph plotted, but on completion of labor, a printout was made for the record and for de-identification before scanning to the study database. The JHSPH and KEMRI Institutional Review Boards required that providers participating in the study give consent but did not require that women cared for during labor provide consent.

## RESULTS

We compared data from 842 clients in active labor in the ePartogram group with 1,042 clients in the paper partograph group.

Table 2 compares the facility characteristics of ePartogram and paper partograph sites, and Table 3 compares the client characteristics. Among facility characteristics, there were nonsignificant differences between paper partograph and ePartogram groups in median number of providers available, volume of deliveries, and proportion of all women that were recruited in the study. Although we used partographs only from study-trained providers, the ePartogram facilities had 94.5% of SBAs trained for the study compared to 65% of SBAs in paper partograph facilities, a significant difference (*P*=.02).

**TABLE 2.** Facility Characteristics by Study Arm, Kisumu and Meru, Kenya, October 2016 to May 2017

	**ePartogram**	**Paper Partograph**	***P* Value**
Number of providers, median (IQR)	12 (8)	9.5 (1)	.24
Study-trained providers at each facility, % (IQR)	94.5 (15)	65 (22)	<.001
Volume of deliveries per facility during study period, median (IQR)	101 (84)	76 (224)	.56
Labors recruited into study, % (IQR)	38.5 (57)	43 (46)	.75

Abbreviations: ePartogram, electronic partogram; IQR, interquartile range.

**TABLE 3.** Client Characteristics by Study Arm, Kisumu and Meru, Kenya, October 2016 to May 2017

	Clients Using ePartogram (n=842)	Clients Using Paper Partograph (n=1,042)	*P* Value
BEmONC facilities, No. (%)	300 (35.6)	317 (30.4)	.02
CEmONC facilities, No. (%)	542 (64.4)	725 (69.6)	.02
Public facilities, No. (%)	842 (100.0)	715 (68.6)	<.001
Faith-based facilities, No. (%)	0 (0.0)	327 (31.4)	<.001
During strike months, No. (%)	439 (53.9)	352 (34.1)	<.001
During non-strike months, No. (%)	376 (46.1)	439 (53.9)	<.001
Parity 0, No. (%)	297 (36.4)	345 (36.6)	.90
Parity 1+, No. (%)	519 (63.6)	642 (63.4)	.90
Admitted during day, No. (%)^a^	496 (58.9)	549 (52.8)	.01
Admitted during night, No. (%)^b^	346 (41.1)	493 (47.3)	.01
Delivered during day, No. (%)^a^	433 (53.1)	542 (52.7)	.80
Delivered during night, No. (%)^b^	382 (46.9)	486 (47.3)	.80
Cervical dilation ≤5 cm at admission, No. (%)	218 (25.9)	371 (36.2)	<.001
Cervical dilation >5 cm at admission, No. (%)	624 (74.1)	642 (63.4)	<.001
Duration of first stage recorded, median (IQR)	120 (180)	555 (360)	<.001

Abbreviations: BEmONC, basic emergency obstetric and newborn care; CEmONC, comprehensive emergency obstetric and newborn care; ePartogram, electronic partogram; IQR, interquartile range.

a Day: 6 am to 6 pm.

b Night: 6 pm to 6 am.

The ePartogram group consisted of public facilities only, with no faith-based facilities, and also had the 2 largest public facilities; 35.6% of recruitment was in BEmONC facilities to 30.4% in the paper partograph group. The study was disrupted by a prolonged health worker strike, during which 53.9% of the recruitment in the ePartogram group compared to 34.1% in the paper partograph group occurred, and this difference was significant. For cervical dilation at admission, 74.1% of ePartogram labors versus 63.4% of paper partograph labors were already beyond 5 cm dilated on admission, which may also partly, but not completely, explain the difference in median duration of the first stage of labor recorded at 120 minutes for the ePartogram group versus 555 minutes for the paper partograph group.

Table 4 compares the proportion of labors in which clinical rules showing abnormalities were triggered. In the paper partograph group, the SBA should recognize an abnormality in clinical rules during ongoing use of the partograph; in the ePartogram group, an abnormality in clinical signs will trigger a visual and an audible alert reminding SBAs to perform measurements when due or to take clinical actions. Clinical rules for fetal well-being (fetal heart rate, moulding, liquor status) were triggered in 43 (5.1%) of ePartograms and seen on 80 (7.7%) of paper partographs; this difference was not significant. Similarly, selected measures of maternal well-being (maternal pulse, temperature, blood pressure, urine protein) were triggered in 242 (28.7%) of ePartogram users, and noted in 338 (32.4%) of paper partographs; again this difference was not significant. However, clinical rules for abnormal labor progress (duration and frequency of contractions) were triggered in 565 (54.2%) of clients in the paper partograph group compared to 191 (22.7%) of clients in the ePartogram group. In this analysis, we excluded clinical rules related to the alert or the action line as those features of the WHO partograph were eliminated in 2018 WHO guidelines. This difference was statistically significant and has been controlled for in the regression analysis.

**TABLE 4.** Clinical Rules Triggered Based on Recorded Parameters Across Type of Partograph Used

	**Clients Using ePartogram, No. (%) (n=842)**	**Clients Using Paper Partograph, No. (%) (n=1,042)**	***P* Value**
Selected measures of fetal well-being triggered (fetal heart rate, moulding, liquor status)	43 (5.1%)	80 (7.7%)	.29
Selected measures of maternal well-being triggered (pulse, temperature, blood pressure, urine protein)	242 (28.7%)	338 (32.4%)	.49
Selected measures of labor progress triggered (duration and frequency of contractions)	191 (22.7%)	565 (54.2%)	.01

Abbreviation: ePartogram, electronic partogram.

Table 5 compares interventions undertaken by providers among clients in the paper partograph and ePartogram groups. Overall, interventions to maintain fetal well-being (position change, oxygen, vacuum extraction, cesarean delivery, or referral) were performed in 116 (14.7%) women in the ePartogram group compared to 54 (5.3%) women in the paper partograph group. This difference was highly significant even when adjusting for differences in the study groups, including effects of the strike period, whether a facility was private, offered CEmONC, the number of providers, parity, and status of cervical dilation at admission (adjusted relative risk [RR]=4.00, 95% CI=1.95–8.19).

**TABLE 5.** Actions Undertaken During Labor by Provider Across Type of Partograph Used

**Intervention**	**ePartogram, No. (%**^a^**)** **(n=842)**	**Paper Partograph, No. (%**^a^**)** **(n=1,042)**	**Crude Relative Risk (95% CI)**	**Crude *P* Value**	**Adjusted Relative Risk (95% CI)** ^b^	**Adjusted *P* Value**
Walk, ambulate	645 (82)	648 (63.3)	1.29 (0.98–1.71)	.07	*Model does not converge*	
Food given	561 (71.3)	456 (44.6)	1.60 (1.14–2.24)	.01	1.73 (1.30–2.30)	<.001
Fluids given	622 (79)	479 (46.8)	1.69 (1.22–2.34)	<.001	1.57 (1.21–2.03)	<.001
Companion present	400 (50.8)	435 (42.5)	1.20 (0.80–1.79)	.39	1.1 (0.70–1.75)	.67
Position of choice	435 (55.3)	255 (24.9)	2.22 (1.05–4.70)	.04	1.49 (0.67–3.30)	.33
Active management of third stage	778 (92.8)	820 (78.7)	1.17 (1.03–1.34)	.02	*Model does not converge*	
Interventions to maintain normal labor	731 (86.8)	697 (66.9)	1.30 (0.96–1.76)	.10	1.09 (0.92–1.29)	.34
Interventions to address fetal well-being	116 (14.7)	54 (5.3)	2.79 (1.03–7.57)	.04	4.00 (1.95–8.19)	<.001
Interventions to addresslabor abnormality	246 (31)	171 (16.7)	1.86 (0.98–3.55)	.06	1.31 (0.67–2.57)	.42

Abbreviation: CI, confidence interval; ePartogram, electronic partogram.

a Percentages exclude missing cases.

b Relative risk adjusted for strike period, CEmONC facility, faith-based facility, number of providers, multiparity, and admission after 5 cm dilatation.

Clients in the ePartogram group were more likely than those in the paper partograph group to have been fed during labor (71.3% versus 44.6%, respectively) and given fluids (79% versus 46.8%, respectively), and these differences were significant even when adjusting for the study group differences listed above. In addition, ePartogram clients were encouraged to ambulate and given active management of the third stage of labor more frequently than clients managed with the paper partograph (82% versus 63.3%, respectively) and (92.8% versus 78.7%, respectively), but this difference could not be evaluated for differences in study samples as adjustment models do not converge. Presence of a companion at birth and position of choice, although more practiced in the ePartogram group than the paper partograph group, were not significantly different when adjusting for group differences. Interventions to maintain normal labor (any of ambulation, food, fluids, companion, and position of choice combined) occurred in 778 (86.8%) clients in the ePartogram group compared to 679 (66.9%) clients in the paper partograph group. However, this difference was not statistically significant when adjusting for the factors listed above.

Interventions generally used to address any abnormal progress of labor (augmentation, cesarean delivery) occurred in 31% of the ePartogram group versus 16.7% of the paper partograph group (adjusted RR=1.31, 95% CI=0.67–2.57), but these differences were not significant. The difference remains nonsignificant when we restricted the analysis to only CEmONC sites (two-thirds of study population). The combined rate of labor augmentation and cesarean delivery was 9.4% in the paper partograph users and 10.1% in ePartogram users. When we restricted analysis to CEmONC sites and only those in which any clinical rule was triggered, the combined augmentation/cesarean delivery rates were 44.4% in the paper partograph group and 30.1% in the ePartogram group.

In Table 6, we explore the effect of ePartogram use versus paper partograph on the secondary outcome of the study, suboptimal maternal outcomes using a log binomial model.

**TABLE 6.** Effectiveness of Electronic Partogram on Maternal Outcomes Using Log Binomial Models (n=1,457)

	**Crude Relative Risk**		**Adjusted Relative Risk**	
	**IRR (95% CI)**	***P* Value**	**Adjusted IRR (95% CI)**	***P* Value**
ePartogram group	0.81 (0.51–1.28)	.36	0.67 (0.36–1.26)	.21
Strike time	0.99 (0.71–1.39)	.96	1.14 (0.90–1.43)	.27
CEmONC facility	1.13 (0.78–1.63)	.52	0.94 (0.61–1.46)	.80
Faith-based facility	1.39 (1.00–1.94)	.05	1.35 (0.94–1.92)	.10
Number of providers	1.00 (0.96–1.03)	.94	1.03 (0.98–1.08)	.28
Multiparity	0.89 (0.76–1.05)	.17	0.91 (0.78–1.06)	.21
Dilation >5 cm	0.90 (0.80–1.01)	.08	0.95 (0.82–1.09)	.44

Abbreviations: CEmONC, comprehensive emergency obstetric and newborn care; CI, confidence interval; ePartogram, electronic partogram; IRR, incident rate ratio.

For suboptimal maternal outcomes, there was no significant difference between the 2 groups after adjusting for facility variables, including disruptions due to the health worker strike, public or private facility, number of providers available at the facility, and client characteristics (parity and cervical dilation on arrival at facility) (adjusted IRR=0.67, 95% CI=0.36–1.26). However, when we restricted analysis only to facilities that offered CEmONC services, we found a significantly lower risk of suboptimal maternal outcomes in the ePartogram group (adjusted IRR=0.15, 95% CI=0.13–0.19).

Table 7 shows the full regression model for the intervention effect of using the ePartogram versus paper partograph for the study’s primary outcome of suboptimal fetal outcomes (even when coexisting with other maternal and labor suboptimal outcomes), after adjusting for facility variables, including disruptions due to the health worker strike, whether it was a CEmONC or BEmONC facility, public or private, number of providers available at the facility, and client characteristics (parity and cervical dilation on arrival at facility and selected measures of fetal and maternal well-being and progress of labor). Overall, the ePartogram group had an adjusted IRR of 0.44 (95% CI=0.27–0.73) for adverse fetal outcomes. In other words, ePartogram use was associated with a 56% (95% CI=27%–73%) lower likelihood of suboptimal fetal outcomes than use of the paper partograph, after adjusting for facility and client-level variables.

ePartogram use was associated with a 56% lower likelihood of suboptimal fetal outcomes than the paper partograph.

**TABLE 7.** Effectiveness of Electronic Partogram on Fetal Outcomes Using Log Binomial Models (n=1,498)

	**Crude Relative Risk**		**Adjusted Relative Risk**	
	**IRR (95% CI)**	***P* Value**	**Adjusted IRR (95% CI)**	***P* Value**
ePartogram group	0.34 (0.22–0.54)	.001	0.44 (0.27–0.73)	.01
Strike time	1.09 (0.77–1.53)	.62	1.33 (0.95–1.88)	.10
CEmONC facility	0.94 (0.45–2.00)	.88	1.13 (0.81–1.57)	.47
Faith-based facility	1.41 (0.83–2.41)	.20	0.80 (0.67–0.95)	.01
Number of providers	0.93 (0.90–0.95)	.001	0.96 (0.93–1.00)	.03
Multiparity	0.67 (0.51–0.88)	.001	0.65 (0.50–0.84)	.01
Dilation >5 cm	0.89 (0.72–1.11)	.31	0.95 (0.82–1.11)	.54

Abbreviations: CEmONC, comprehensive emergency obstetric and newborn care; CI, confidence interval; ePartogram, electronic partogram; IRR, incident rate ratio.

Because we allocated the 2 largest CEmONC facilities to the ePartogram group and randomly assigned the other 10 facilities to intervention or control, we ran the full regression model restricting only to the 10 randomly assigned sites (4 BEmONC facilities for ePartogram, 4 CEmONC and 2 BEmONC facilities for paper partograph). On this subset, the ePartogram group had an adjusted RR of 0.33 (95% CI=0.22–0.52) for suboptimal fetal outcomes. In other words, ePartogram use in smaller facilities was associated with a 67% (95% CI=48%–78%) lower likelihood of suboptimal fetal outcomes than use of the paper partograph, after adjusting for facility and client-level variables, confirming an even greater effect size than in the total sample.

In Table 8, we present the only before and during study comparison of ePartogram and paper partograph sites for early perinatal mortality estimates based on monthly facility register data during the 6 months before the study compared to facility data during the study period. The outcome of interest (ratio of stillbirths and neonatal deaths in the first 24 hours to total births) is referred to here as early perinatal mortality (EPM). All the EPM recorded in facility registers during the 6 months before the study and during the study regardless of study recruitment status are analyzed here. In ePartogram facilities, the incidence of EPM per 100 births decreased from 2.21 (95% CI=1.77–2.72) in the pre-intervention period to 1.82 (95% CI=1.49–2.20) during the intervention period, giving an adjusted IRR of 0.81 (95% CI=0.66–1.01; *P*=.06). In paper partograph sites, the incidence of EPM per 100 births increased from 2.82 (95% CI=1.99–3.99) before the study to 3.05 (95% CI=2.29–4.05) during the study, giving an adjusted IRR of 1.12 (95% CI=0.89–1.41; *P*=.34).

**TABLE 8.** Effect of the Electronic Partogram Intervention on Incidence of Early Perinatal Mortality^a^ Using Facility Data

	EPM Incidence per 100 Births^b^ (95% CI)	EPM Incidence per 100 Births^c^ (95% CI)	Adjusted IRR^d^ (95% CI)	Difference-in-Difference (95% CI)	*P* Value
ePartogram	2.21 (1.77–2.77)	1.82 (1.49–2.20)	0.81 (0.66–1.01)	0.73 (0.53–1.00)	*P*<.05
Paper partogram	2.82 (1.99–3.99)	3.05 (2.29–4.05)	1.12 (0.89–1.41)		

Abbreviations: CI, confidence interval; ePartogram, electronic partogram; EPM, early perinatal mortality; IRR, incident rate ratio.

a Early perinatal mortality comprises neonatal (<24 hours) deaths + fresh stillbirths. Births comprise live births + fresh stillbirths. This analysis included all EPM recorded in the health facility, regardless of whether enrolled in study or not. The model accounts for facility-level clustering and adjusts for facility variables of affiliation (public versus private), capacity to address emergencies (CEmONC versus BEmONC), presence of a medical or clinical officer (yes/no), percentage of providers trained in labor management by study staff (≥75% or <75%), median years of provider experience, and whether a health worker strike was occurring in the month in public facilities (yes/no).

b Six-month period before study.

c During study implementation.

d Comparing before and during study rates in each group.

The ratio of IRRs comparing the change in rates of EPM in ePartogram sites to paper partograph sites before and after implementation, after adjusting for baseline facility characteristics, was 0.73 (95% CI=0.53–1.00; *P*=.049). This suggests that the reduction of EPM in ePartogram sites is trending in the favorable direction compared to paper partograph sites.

Table 9 explores compliance to a set standard for frequency of observations as recorded on the ePartogram or paper partograph. Examination of every 30-minute period of recorded observations showed that the ePartogram recorded all measurements, except for fetal heart rate and contractions, more frequently than the paper partogram. Of the 3 measurements to be recorded every 30 minutes (fetal heart rate, contractions, maternal pulse), maternal pulse was more likely to be recorded on the ePartogram than the paper partograph. All urine measurements (volume, acetone, protein) were recorded more frequently on the ePartogram.

**TABLE 9.** Compliance With Recommended Frequency of Recording of Observations During Labor

**Measure**	Instances When Observation Could Be Recorded, %		Instances When Observation Should Have Been Recorded Per Standard, %	
	**ePartogram**	**Paper Partograph**	**ePartogram**	**Paper Partograph**
**Recommended timing:**	**Every 30 minutes**		**At least every 1 hour**	
Fetal heart rate	76.0	84.0	80.2	85.9
Contractions	76.0	82.0	79.5	90.7
Maternal pulse	72.0	42.0	73.9	33.8
**Recommended timing:**	**Every 2 hours**		**At least every 2 hours**	
Temperature	43.0	18.0	99.3	72.6
**Recommended timing:**	**Every 4 hours**		**At least every 4 hours**	
Color of amniotic fluid	43.0	23.0	100.0	65.8
Moulding	42.0	18.0	100.0	60.1
Cervical dilatation	51.0	35.0	100.0	99.8
Descent	50.0	34.0	99.7	98.0
Blood pressure	42.0	25.0	99.7	92.2
Urine protein	32.0	5.0	95.8	29.0
Urine acetone	33.0	5.0	96.1	28.5
Urine volume	33.0	5.0	96.4	31.6

Abbreviation: ePartogram, electronic partogram.

In terms of compliance to measurement frequency norms, only a small fraction of each group was fully compliant for measures to be done every 30 minutes. ePartogram users were more compliant than paper partograph users in measuring pulse, temperature, amniotic fluid status, moulding, blood pressure, and urine; both groups were equally compliant in recording cervical dilation and descent of the presenting part; and paper partograph users were more compliant with recording fetal heart rate and contractions.

### Qualitative Interviews

Feasibility and acceptability of the ePartogram technology were examined during in-depth interviews. SBAs noted minimal issues with care and maintenance of the tablet and were able to keep the devices clean. Some electricity outages were noted, but SBAs reported no issues with charging tablet devices.

SBAs unanimously agreed that once they were trained with the ePartogram, data entry was simple and user-friendly. Several SBAs cited issues with the inability to correct ePartogram entry errors after 15 minutes had elapsed. If a parameter was entered incorrectly and not caught in time, the system would continually alert the SBA to a nonexistent concern. The SBAs found this distracting.

SBAs also noted challenges with ePartogram data entry when caring for multiple clients.

*Like maybe when you are entering the data for a mother who has just come, in regard to the time you are entering the fetal heart, the contractions, the dilations so after you are through with the mother and now you want to enter [data], you find there have a difference of 1 minute, 1 minute, 1 minute so assuming you have 3 or 4 mothers then it will reach the due time for entering the next data; that difference for 1 minute for 3 or 4 mothers, it is hectic*. —SBA at a health center

The ePartogram emits audible reminders when it is time for an SBA to take a clinical measurement. Inputting an abnormal clinical value triggers an alarm. SBAs noted that the reminders were clear and helpful, and they especially appreciated that the ePartogram dashboard within the application highlighted when to take measurements and which measurements were overdue. In-charges also noted that the ePartogram improved real-time measurement recording and reduced retrospective data entry.^17^ SBAs reported that having reminders increased the frequency with which they checked clients and prompted them to take clinical action when needed compared to use of the paper partograph.

*For me, it is just the quality of work, because for the paper partograph, you just leave it … until when after the mother delivers you fill in what you had not filled in, but for (the ePartogram) you have to be on toes so that if there is a problem or some necessary intervention, it just shows you immediately instead of waiting for a delay that maybe will result to other unwanted things like fetal distress, maternal distress.* —SBA at a health center

SBAs made clear that the ePartogram was not a panacea for all clinical decision making challenges and that attitudes and behavior change must also be addressed.

*[T]he device will only give you an alert and if you are asleep or you are not acting on what next needs to be done, the alerts will continue and the outcome will be negative, so if we also need behavior change, that would be good.* —Hospital in-charge

Alerts also prompted action for urinalysis for protein.

*There are some of the things that have not been done routinely previously. But currently, the device is helping us to do, like, the urine test. . . . [N]owadays every client has to be tested. Before we would do the urine test only when we realize the blood pressure is elevated, but today the device will not allow you to continue. It will keep on ringing [an] alarm, so you have no option but to do it.* —Hospital in-charge

SBAs were overwhelmingly pleased with the technology, but raised concerns about introducing the ePartogram at high-volume sites. SBAs often noted that once the facility reached around 4 clients per SBA, timely data entry was difficult. Additionally, they suggested that other team members be trained to use the ePartogram (e.g., surgical staff who take over care once a client gets to the operating room, as well as students since they often monitor clients). SBAs were concerned that failure to act or respond to an alert due to staff shortage or more pressing complications with other clients may be recorded in the ePartogram and viewed as neglect. However, despite the challenges, SBAs agreed that the ePartogram was a great improvement over the paper partograph, even at high-volume sites.

*Because my experience is that ePartogram helps us to manage labor better. In busy facilities, it becomes difficult to enter all that information on the paper partograph because paper takes a lot of time before you fill, but it is easier with ePartogram.* —SBA at a health center

SBAs were pleased with the ePartogram but raised concerns about using it at high-volume sites because timely data entry with more clients might be difficult.

Supervisors noted that ePartogram improved their ability to oversee both their subordinates and patients at both a clinical and management level.

*The supervisor application is good in such a way that I have it all the time, I can monitor what work is going on in the labor ward if there is something that needs my attention. I could call the nurse on duty and she would check and see so the supervisor’s application is there to support the nurse on the ground.* —Physician supervisor at a large hospital

The ePartogram provides real-time sharing of labor observations, enabling on-site and off-site supervisors, as well as SBAs at referral facilities, to view client information and provide consultation as needed. Supervisors noted that this feature improved their ability to oversee their subordinates and clients on both a clinical and managerial level. They also noted that the ePartogram improved communication in both directions and that SBAs used the supervisory communication system to voice concerns about clients and ask questions.

*[W]henever there is a need I can always come very fast from wherever I am. I am aware what is happening in [the] labor ward. If I am outside the hospital, I am aware [of] what is happening in [the] labor ward and we have kept that continuous conversion.* —Hospital in-charge

For SBAs in referral facilities, the ePartogram allowed surgical teams to better prepare for incoming referral clients and to communicate in a network. Although most facilities communicated by phone during referrals when using the paper partograph, the ePartogram provided a more detailed account of the client’s labor and complications. One SBA working in a large facility hypothesized that a shift in the number of cesarean deliveries in her facility was due to early identification and management of complications.

*Yes, when we are using ePartogram in our labor ward, the C-section rate goes down, the number of complications goes down because they are doing early interventions and you are able to bring everybody on board so that now, it is a team work approach, the picture is better. I am now imagining [if] other facilities are using it, we will actually cause a paradigm shift about maternal health care in the system.* —Hospital in-charge

## DISCUSSION

Our study is one of the first to test the feasibility and effectiveness of using an electronic decision-support tool for intrapartum care in limited-resource settings. The results of this quasi-experimental study demonstrated the effectiveness of SBA use of an electronic partograph on an Android tablet. The ePartogram’s use was associated with improved fetal outcomes compared to use of paper partographs, and the intervention was well accepted by care providers and their supervisors. Furthermore, use of the ePartogram was associated with a trend in a decline in EPM after the introduction of ePartogram compared to the preceding 6 months when the facilities used the paper partograph. To our knowledge, this is the first report on the effectiveness of a digital labor decision-support system in limited-resource settings.

The ePartogram’s use was associated with improved fetal outcomes compared to paper partographs, and the intervention was well accepted by care providers and their supervisors.

In Kenya, as in most limited-resource countries, SBAs monitor labor using the paper partograph while providing care to clients to maintain normal labor, manage complications, and assist delivery. Often, the task of monitoring labor is delegated to junior midwives or students, with senior SBAs checking in periodically. Ideally, monitoring fetal and maternal well-being and labor progress will enable SBAs to identify or predict complications immediately and take appropriate actions, resulting in improved maternal and fetal outcomes. In our study, we reinforced labor monitoring and good routine care norms and protocols in both groups during a 2-day refresher training but did not attempt to judge whether the actions taken during the study were appropriate to the problem identified by the monitoring process. For those using the ePartogram, the additional input was entering observations in the tablet device that gave audible alerts if a measurement was not taken on time and visual alerts when a single or a combination of more observations were determined to be abnormal or predicted a potential complication. SBAs valued the ePartogram’s audible and visual alerts, which allowed them to spend more time determining what might be wrong and why, rather than if something was wrong.

Our study found that the ePartogram group had lower rates of clinical rules triggered, significantly so for duration and frequency of contractions. One explanation is that with an ePartogram, an audible sound alerts the provider and can also be seen by the supervisor, so actions are more likely to be promptly taken to correct the situation. With the paper partograph, a provider may fail to recognize that a clinical rule has been triggered and actions may be delayed, potentially setting off another clinical trigger. In-depth interviews with supervisors indicated that they were proactive when they are also being alerted in real time by the app rather than awaiting to be called, as with the paper partograph. This would be an important area to research further. One concern is that providers may get alarm fatigue and choose to ignore auditory alerts.

As technology use in labor rooms increases, there is some concern that personal attention and care will suffer.^29^ Our study showed that clients in the ePartogram group were more likely to receive care to maintain normal labor, including encouragement to ambulate, feed, drink fluids, and receive active management of the third stage of labor.

One challenge with the paper partograph is that it is sometimes used solely for recordkeeping rather than decision making, and labor measurements are often entered retrospectively, which the ePartogram does not allow.^11^^–^^14^^,^^17^ Although our study showed that fetal heart rate and contraction measurements were recorded more frequently in the paper partograph group, we did not evaluate whether or not monitoring frequency was exaggerated in some cases or if some of these were entered retrospectively.

It was noteworthy that users of the ePartogram found that taking care of 4 or more patients limits the feasibility of use or usefulness of the ePartogram. We do not believe that the ePartogram should be used to take care of more patients than used for paper-based labor charts. However, time-saving features, such a drop-down menus and auto plotting, make it easier to do the right thing and providers have more time to provide respectful care, perform interventions, and perform observations on time, all of which would improve outcomes.

Interviews confirmed the ease of entry with the ePartogram, and ePartogram users appreciated the drop-down menus, as also noted by Litwin et al.^22^ Further, interviewed in-charges and SBAs stressed that the ePartogram prompted action, and they discussed specific examples of the ease of use during labor management and decisions to refer the laboring client or operate.

The observation by a hospital supervisor that he had observed lower cesarean delivery rates warrants further analysis, but our data indicate that at CEmONC sites and among patients who had any clinical rule triggered, the combined labor augmentation/cesarean deliveries are lower in ePartogram-managed labors, but our study was not powered sufficiently to attribute this to ePartogram use. However, we found that suboptimal maternal outcomes were significantly lower when the ePartogram was used in CEmONC facilities.

Unfortunately, the rate of cervical dilation has dominated decision making instead of using the combination of measures of fetal and maternal well-being and progress of labor, all of which are visually represented in the paper partograph. The ePartogram depends on clinical rules that place emphasis on all these measurements, not just alert and action lines. The latest WHO guidelines and many clinicians have questioned Friedman’s labor curve. Of the 77 clinical rules that form the decision-support part of the ePartogram, only 3 are related to the rate of progression of cervical dilation and the alert/action line. Another 4 are related to the action line but in relation to number and duration of contractions and descent of presenting part.

The ePartogram is based on clinical rules that were developed and validated between 2013 and 2016 using WHO’s guidance^30^ at that time and clinical consultation. Since then, WHO completed a large prospective cohort study^31^ and has issued new recommendations for intrapartum care for a positive childbirth experience.^32^ By far the most consequential new recommendations relate to definitions of onset of the first stage of labor. First stage is now defined as regular painful contractions, substantial effacement, and more rapid dilation from 5 cm. The active phase usually does not exceed 12 hours in first labors and 10 hours in subsequent labors. In spontaneous labor, cervical dilatation of 1 cm per hour during active phase, as depicted by the alert line, does not accurately identify women at risk of adverse birth outcomes, and a minimum cervical dilation of 1 cm per hour is unrealistic for some women. WHO is developing a new partogram; however, its adoption will likely require a massive effort, including efforts to replace the existing paper partograph. The ePartogram’s clinical decision-support system consists of 77 clinical rules, 8 of which will be changed as a result of the new guidelines. We believe that a digitally connected app like the ePartogram can accelerate adoption of the new guidelines at scale.

In 1955, Friedman published the labor progression curve that drove obstetric practice for more than 40 years.^33^ Perhaps less known is that his conclusions were based on a study involving only 500 women with an average age of 20 years. Fifty-five percent had forceps delivery; 9% underwent cesarean delivery; and 22% received “twilight sleep,” 42% with moderate sedation and 31% with heavy sedation.^33^ By no stretch of the imagination can we call that a “normal” population to define normal labor or create labor cervicographs. Yet we continued to use Friedman’s curve well into this century. In 2002, Zhang reassessed the labor curve in nulliparous women,^34^ and in 2013, Boyle et al. examined 38,484 first-time cesarean deliveries, of which 30.8% were done for primigravidas, 31% were due to “failure to progress,” and 40% were for women less than 5 cm dilated.^35^ They concluded that more than 10% of primigravidas have unplanned and unnecessary cesarean deliveries due to “failed progress” in early labor. These studies have been confirmed in Asia, Latin America, and most recently in Africa.^31^ The ePartogram digitizes labor data and holds it in a central server. Digitizing labor measurements and including prenatal, outcome, and postnatal data offers the exciting possibility of harnessing artificial intelligence and machine learning principles to further refine clinical rules and enhance clinical decision-support in a manner that has never been possible before without relying on sample studies.^36^^,^^37^

In our analysis of measures of labor progress, we excluded cervical dilation and only included number and duration of contractions and descent of presenting art, in recognition of new WHO guidelines and the many studies that confirm that Friedman’s original assertion of 1 cm per hour is flawed. One additional value of a digital labor chart is in our ability to add or eliminate rules as they are formulated or as new evidence dictates.

The challenges of introducing a digital labor device include cost, cybersecurity, and equipment malfunction. Although the unit cost of tablets has declined, other cost implications exist. Smartphones are ubiquitous and less expensive. Furthermore, clinical workflows may need to be adapted to ensure that the ePartogram is implemented efficiently. For example, in the larger facilities, SBAs teach students how to use the paper partograph, so it was challenging for them to teach the paper partograph while using the ePartogram on their clients. Against these challenges were the ability of supervisors to access labor data in real time and the existence of complete digital records. Therefore, a cost-benefit analysis is needed. A major challenge to overcome is building a sufficiently robust central server to host the vast amount of labor data being generated in real time.

Challenges of introducing a digital labor device include cost, cybersecurity, and equipment malfunction.

An adapted version of the ePartogram is being tested in India on a larger scale. It uses prevailing labor charts in India and also digitizes other pregnancy data and outcome data. We hope to align this version with new WHO guidance by altering 8 of the 77 clinical rules and using the newly developed WHO labor charts that are undergoing testing right now. In addition, secondary analysis of data will reveal what proportion of interventions are warranted and not warranted by adjusting these clinical rules. We believe future research should focus on rationalizing the clinical rules so as to prevent alert fatigue and evaluate with more robust design the supervisor function. An important area to explore further is compliance to set standards of observations especially when interventions, such as augmentation, are performed. Finally, there is a potential to harness the growing volume of digitized labor, intervention, and outcome data for machine learning and artificial intelligence.

### Study Limitations

An important limitation to our study is that the ePartogram group and paper partograph group were not similar in a number of key characteristics, including the fact that the 2 largest public facilities were in the ePartogram group and the 2 faith-based hospitals were in the paper partograph group. The study was disrupted by a prolonged health worker strike, the effect of which may have been unevenly distributed among sites and probably led to a greater proportion of patients arriving much later in labor than we had expected. We have addressed these differences by adjusting for them in all outcome analyses.

One design limitation was that the 2 largest facilities were allocated to the ePartogram arm and the other 10 smaller facilities were randomly distributed. Our analysis of only these 10 randomly assigned sites showed an even bigger effect size for the primary outcome of suboptimal fetal outcome than when we included all 12 facilities in our analysis.

One limitation was that a greater proportion of patients in the ePartogram group came after 5 cm dilation than in the paper partograph group and that median duration of labor recorded on paper partographs was much longer than on the ePartogram. One possible explanation is that the ePartogram did not allow for retrospective entry of data after a certain period of time (30 minutes after the parameter recording was due) but such entries were possible on the paper version. Another possible explanation is that providers may be waiting longer to start the ePartogram than with paper partographs, but this was not raised during our in-depth interviews. We do, however, show that while there was a longer period of labor observed in the paper partograph, there was much poorer compliance to labor observation norms.

The study sample facilities are heterogeneous, with different levels of care and management, and the distribution across the ePartogram and paper partograph groups differed.

The information recorded was not always complete and differed by ePartogram and paper partograph groups, possibly resulting in a differential bias across the groups. In Table 8, we show that except for 2 parameters (fetal heart rate and contractions,), every other observation is better recorded in the ePartogram group, and there is, in fact, less missing recording of observations in the ePartogram, perhaps as a result of the “nag” feature that alerts a provider and their supervisors that observation are overdue. The data collected as part of the study are entirely representative of the routine status of patient records. Methods to assess missingness assume that the missing data are random or one is able to understand and explain the pattern. Hence, we chose not to use any of these methods, as they would obfuscate the data.

SBAs in the study may have based their decision to start a client on the ePartogram, paper partograph, or not start a partogram at all on criteria such as staffing and number of clients under observation for labor. Many clients probably arrived in very late stages of labor so there was not time to record more than 1 set of parameters (at admission). We are unable to completely explain the processes that went into which woman was put on the partographs and what data were entered. These conditions, which differed by facility, may have resulted in a selection bias in the sample of women, and this bias may be difficult to quantify in direction or magnitude. Thus, the ePartogram was implemented within a clinical setting where both the ePartogram and paper partographs were used, and during this introductory phase, not all clinical workflows were updated to accommodate ePartogram use.

An additional limitation is interpreting the reduction in early perinatal mortality in ePartogram sites because we provided refresher training to a greater number of SBAs in those sites than in paper partograph sites even though we have adjusted for that difference. Also, our training of SBAs that included reinforcing recordkeeping occurred during the 6 months before the intervention, and it is possible that records were better in the period after training than in the pre-intervention period. We do not think this limits conclusions from our other analysis as we only included labor charts from study-trained SBAs for all outcomes.

Data collection was delayed (particularly in public-sector facilities) due to a health worker strike in both Meru and Kisumu counties. The ePartogram application performance and speed slowed after 1 to 2 months of clinical use, and updated software was deployed to address these challenges. The study’s main conclusion of improvement in fetal outcomes among ePartogram users remains significant even after adjusting for facility characteristics.

The differences between the groups may (and would) have resulted in the observation of differences in the outcome. However, not all the effect size can be explained by the baseline differences alone. The intervention group may have been subjected to greater scrutiny and more frequent supervision due to the use of a novel technology. The novelty of tablets alone is likely to improve the attention of providers and contribute to better outcomes. Given the drawbacks in the design of the study, we feel that the effect size should not be the only evidence of impact but placed in the context of the rest of the data.

## CONCLUSION

Use of the ePartogram resulted in significant improvements in fetal outcomes and use of interventions to maintain normal labor compared to the paper partograph. SBAs using ePartograms were also at least as or more compliant in recording measurements during labor compared to SBAs using paper partographs—they noted that the alerts prompted them to take clinical action. As WHO develops a revised partograph that reflects its 2018 intrapartum care recommendations, the ePartogram has great potential to improve quality of care and outcomes through consistent and meaningful labor monitoring and clinical care.

## Notes

**Peer Reviewed**

**Funding:** The study was funded by Jhpiego, an affiliate of Johns Hopkins University, and GE Foundation. Ongoing ePartogram app adaptation and maintenance was funded by GE Foundation and Laerdal Foundation. The GE Foundation and Laerdal Foundation had no role in study design, data collection, data analysis, data interpretation, or manuscript development.
